# Cryptogenic hepatic insult, failing heart and advancing age: a case report

**DOI:** 10.1186/1757-1626-1-408

**Published:** 2008-12-19

**Authors:** Akansha Agrawal, Manish Soneja, Ashish Goel, H Pati, Aparajit B Dey

**Affiliations:** 1 Department of Medicine, All India Institute of Medical Sciences, ND 110029, India

## Abstract

**Background:**

Weakness and fatigue are accepted as normal accompaniments of aging. Usually, older individuals are not investigated with much enthusiasm but a treatable cause is discernible on several occasions.

**Case presentation:**

We had a 67 year old hypertensive lady with a mitral stenosis, presenting in ischemic or hypertensive heart failure with underlying valvular disease, without pulmonary hypertension in sinus rhythm. She had pancytopenia with severe anemia and raised liver enzymes. Bone marrow examination showed aplastic anemia. She was treated with ATG and improved subsequently to become transfusion free. However, she succumbed to an unrelated sudden cardiac death.

**Conclusion:**

Our patient is unique in her uncommon presentation, complex management issues and a favorable outcome after a long and persevering therapeutic intervention and finally her sudden death.

## Case report

In December, 2007 a 67 year old hypertensive lady, with a known rheumatic mitral stenosis, presented with insidious onset, gradually progressive fatigue of one month, which had decompensated acutely. There was no history of peptic ulcer, use of NSAIDs or change in bowel habits. She had no previous blood transfusions or jaundice. She denied smoking or taking alcohol. There was no suggestion of long standing liver or kidney disease, diarrhea or infection. She took amlodipine and atenolol for hypertension. There was no history of intake of any other drugs that could have had a toxic potential.

She was pale, anicteric, normotensive and tachypneic with pulse of 100/minute. Her neck veins were engorged and she had pedal edema. She was afebrile, did not have any clubbing, and had no signs of rheumatic activity or infective endocarditis. She had a loud first heart sound, a normal second heart sound, an opening snap and a mid-diastolic murmur at apex. She had a resonant percussion note, equal air entry, and vesicular breath sounds on both sides. Coarse rales were heard in the infrascapular and infraaxillary areas. She had an enlarged tender, firm liver with sharp margins and a span of 10 cms. No splenomegaly or ascites were noted. Here, we had an old hypertensive lady with a mitral stenosis, presenting in ischemic or hypertensive heart failure with underlying valvular disease, without pulmonary hypertension, rheumatic activity or infective endocarditis in sinus rhythm. An infective pathology causing acute deterioration or a pulmonary embolism was also considered.

**Figure 1:**
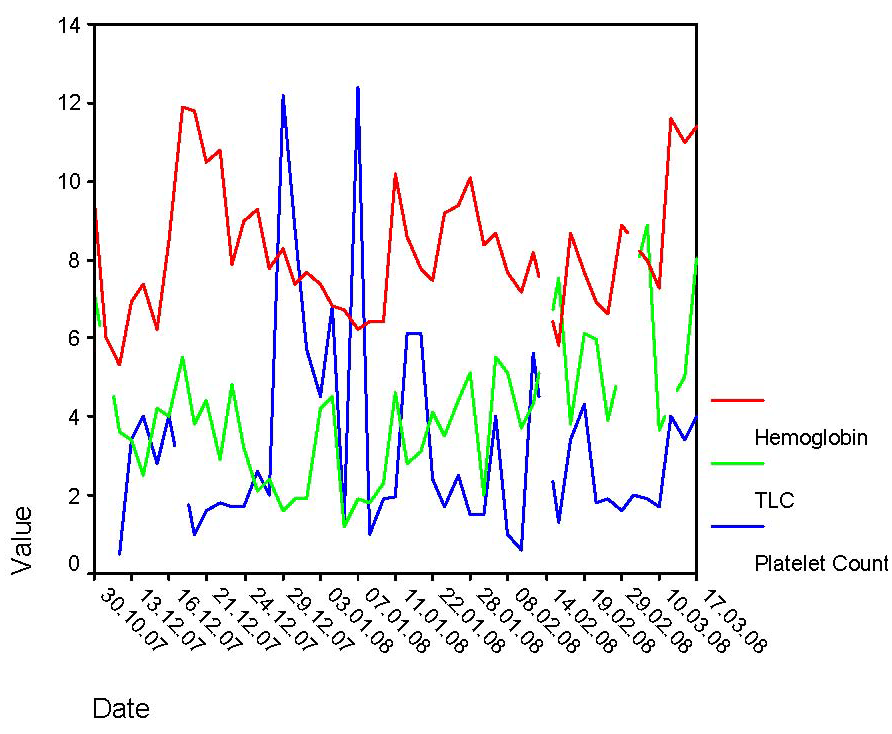
**Distribution of hemoglobin level, platelet count and total leukocyte counts over time**. To represent comparative patterns of platelets, hemoglobin and leukocyte count in the same figure, the platelet count has been represented as actual count * 10^-4^, leukocyte count has been represented as actual count * 10^-3 ^and the hemoglobin has been represented as its actual value.

Her hemoglobin was 65 g/L, TLC 3.2 × 10^9^/L with absolute neutrophils count of 1.6 × 10^9^/L, platelet count of 43 × 10^9^/L and MCV 110 fL (see figure 1). Macrocytosis, hypochromia, leukopenia and reduced platelets but no abnormal cells were seen. Blood cultures were sterile. She had raised serum bilirubin (2 mg/dL) and liver enzymes (SGPT 877 and SGOT 1179 IU). Serology for hepatitis virus A, B, C, E and HIV was negative. Coombs test and antinuclear antibodies were negative. Serum vitamin B_12 _level was 896 pg/ml.

Echocardiography showed mild mitral stenosis (MVOA 1.8 sqcm) with normal left ventricular ejection fraction, and no signs of infective endocarditis. Serum and urine electrophoresis did not detect any abnormal bands. Bone marrow biopsy from the iliac crest showed profound hypoplasia with overall cellularity of less than 5%. She had osteoporosis with a bone mineral density of 0.714 g/sqcm. Her liver function tests rapidly returned to normal levels.

She was given immunosuppression with anti-thymocyte globulin at the dose of 40 mg/kg daily for 4 days along with prednisolone at 1 mg/kg tapered over 3 weeks. This was followed by cyclosporine at the dose of 10 mg/kg/day. The patient continued to need regular and frequent blood and component transfusion support for ten more weeks. The profile of her hematological parameters over this period is reflected in table 1. When seen in April, she was doing well and had been transfusion free for three weeks. She subsequently remained free of complications for another month, but then she complained of acute abdominal pain and succumbed before she could be taken to the hospital for medical attention suspected to have had an unrelated sudden cardiac death. A post mortem examination could not be performed.

**Table 1:** Progression of the hematological parameters of the patient over time

**Date**	**Hemoglobin**	**Platelet Count**	**Total Leukocyte Count**
30.10.07	9.6	.	7200

11.12.07	6	.	.

12.12.07	5.3	5000	3620

13.12.07	6.9	34000	3400

14.12.07	7.4	40000	2500

15.12.07	6.2	28000	4200

16.12.07	8.5	40000	4000

19.12.07	11.9	.	5500

20.12.07	11.8	10000	3800

21.12.07	10.5	16000	4400

22.12.07	10.8	18000	2900

23.12.07	7.9	17000	4800

24.12.07	9	17000	3200

26.12.07	9.3	26000	2100

27.12.07	7.8	20000	2400

29.12.07	8.3	122000	1600

31.12.07	7.4	88000	1900

14.01.08	8.6	61000	2800

18.01.08	7.8	61000	3100

22.01.08	7.5	24000	4130

24.01.08	9.2	17000	3500

27.01.08	9.4	25000	4400

28.01.08	10.1	15000	5100

31.01.08	8.4	15000	2000

02.01.08	7.7	57000	1900

15.02.08	5.8	13000	7540

17.02.08	8.7	34000	3800

19.02.08	7.7	43000	6130

23.02.08	6.9	18000	5970

26.02.08	6.6	19000	3920

29.02.08	8.9	16000	.

03.01.08	7.4	45000	4200

03.03.08	.	20000	.

14.03.08	11	34000	5000

17.03.08	11.4	40000	8040

04.01.08	6.8	68000	4500

04.02.08	8.7	40000	5500

06.01.08	6.7	13000	1200

07.01.08	6.2	124000	1900

07.03.08	8	19000	8900

08.02.08	7.7	10000	5100

09.01.08	6.4	10000	1800

09.02.08	7.2	6000	3700

10.01.08	6.4	19000	2300

10.03.08	7.3	17000	3670

11.01.08	10.2	19500	4600

11.02.08	8.2	56000	4320

12.03.08	11.6	40000	.

Weakness and fatigue are accepted as normal accompaniments of aging. Usually, older individuals are not investigated with much enthusiasm but a treatable cause is discernible on several occasions. Here, anemia with CHF was evident at presentation. Chronic disease, iron deficiency, vitamin B_12 _or folate deficiency, gastrointestinal bleeding and myelodysplastic syndrome are commonly identified.[1] Aplastic anemia, remains rare in older persons. Older patients are usually ineligible for allogeneic bone marrow transplantation, owing to absence of a donor, advanced age and frail phenotype. Immunosuppression with cyclosporin and antithymocyte globulin (ATG) is often contemplated but infrequently tried in older individuals.[2,3] It is known that 50% younger patients respond within 3 months of immunosuppression, and about 75% by 6 months, and become transfusion independent, but some may have a persistently hypoproliferative marrow. In the current case, bone marrow suppression followed a transient cryptogenic hepatitis, likely of a viral etiology. Aplastic anemia has been reported between 3–6 months following cryptogenic hepatitis in younger patients.[4,5] A more protracted course might be seen in older patients following immunosuppression. Our patient is unique in her uncommon presentation, complex management issues and a favorable outcome after a long and persevering therapeutic intervention and finally her sudden death.

## Consent

Consent could not be taken from the patient before publication because she expired before this could be done. Care has been taken to preserve the confidentiality of patient identity

## Competing interests

The authors declare that they have no competing interests.

## Authors' contributions

AA and MS were involved with the day to day management of the patient. AG supervised patient management and completed the final draft of the manuscript. HP was involved in coordinating the pathology reports and arriving at a diagnosis for the patient. ABD held the over all responsibility of patient care and took the final decisions regarding management
